# Case Report: ^18^F-FDG PET/CT findings and SUVmax characterization in a dog with apocrine gland anal sac adenocarcinoma

**DOI:** 10.3389/fvets.2026.1977879

**Published:** 2026-10-16

**Authors:** Bohyeon Shim, Yujin Kim, Sungin Lee

**Affiliations:** Department of Veterinary Surgery, College of Veterinary Medicine, Chungbuk National University, Cheongju, Republic of Korea

**Keywords:** ^18^F-fluorodeoxyglucose, apocrine gland anal sac adenocarcinoma, case report, positron emission tomography, standardized uptake value

## Abstract

Apocrine gland anal sac adenocarcinoma (AGASACA) is a malignant epithelial tumor in dogs that commonly metastasizes to regional lymph nodes. Although ^18^F-fluorodeoxyglucose (^18^F-FDG) positron emission tomography/computed tomography (PET/CT) has been mentioned as a staging modality for this tumor, detailed imaging findings and maximum standardized uptake values (SUVmax) have not previously been reported. A 10-year-old castrated male Maltese was referred for a right anal sac mass and suspected lymph node metastasis. Preoperative ^18^F-FDG PET/CT revealed increased uptake in the primary mass (SUVmax 7.3), the internal iliac lymph node (SUVmax 6.35), and the lumbar aortic lymph node (SUVmax 4.3), with no evidence of distant metastasis. Therefore, the dog was therefore classified as Stage IIIa. Based on these findings, intra-abdominal lymphadenectomy and right anal sacculectomy were performed. Histopathology confirmed AGASACA in the primary mass and morphologically similar metastatic adenocarcinoma in the resected lymph nodes. To our knowledge, this is the first report of ^18^F-FDG PET/CT findings and SUVmax values in a dog with AGASACA and histologically confirmed lymph node metastases. These findings suggest that PET/CT may provide complementary metabolic information for preoperative nodal staging.

## 1 Introduction

Apocrine gland anal sac adenocarcinoma (AGASACA) is a malignant epithelial neoplasm arising from the anal sac, accounting for approximately 2% of all cutaneous tumors and 17% of perianal malignancies in dogs (1). Affected patients typically present with clinical signs attributable to mechanical obstruction caused by the primary tumor or metastatic lymph nodes including tenesmus and constipation, as well as systemic manifestations of paraneoplastic hypercalcemia. The mainstay of treatment for AGASACA is surgical resection of the primary tumor, with concurrent lymphadenectomy recommended when locoregional lymph node metastasis has been confirmed (2).

^18^F-Fluorodeoxyglucose (^18^F-FDG) positron emission tomography/computed tomography (PET/CT) is a hybrid imaging modality that simultaneously provides anatomical and functional metabolic information using the radiopharmaceutical ^18^F-FDG (3). Malignant and inflammatory tissues exhibit upregulated glucose metabolism, resulting in increased ^18^F-FDG uptake that is visualized as areas of intense signal on PET images. The degree of ^18^F-FDG uptake is quantified using the standardized uptake value (SUV), calculated by dividing the tissue ^18^F-FDG concentration (MBq/mL) by the injected activity per unit body weight (MBq/g) (4). In human oncology, ^18^F-FDG PET/CT has become an established staging tool with demonstrated superiority over CT alone in evaluating disease extent across multiple tumor types (5). In veterinary medicine, it has also been investigated in several distinct tumor types, including lymphoma, osteosarcoma, mammary carcinoma, cutaneous squamous cell carcinoma, and granulosa cell tumor (6–9). However, its utility for diagnosis and staging in specific tumor entities remains limited.

Although ^18^F-FDG PET/CT has been mentioned as one of the staging modalities employed in AGASACA, detailed imaging findings and SUV measurements have not been previously reported (10). This report describes the preoperative PET/CT findings and SUVmax values of the primary anal sac tumor and metastatic lymph nodes.

## 2 Case description

A 10-year-old castrated male Maltese weighing 3.88 kg was referred for further evaluation of a right anal sac mass and intra-abdominal lymphadenomegaly. The dog had been castrated at 1 year of age. Based on its anatomical location, the mass was suspected to be an anal sac tumor. Fine-needle aspiration (FNA) of the mass, performed at the referring institution, revealed neoplastic cells with cytological features of malignancy. No preoperative tissue biopsy was performed, and the definitive diagnosis could not be made until postoperative histopathological examination. The patient was subsequently referred for ^18^F-FDG PET/CT to characterize the mass and assess for lymph node metastasis.

Physical examination revealed bilateral popliteal lymphadenomegaly measuring 5 mm, as well as enlargement of the left prescapular lymph node measuring 10 mm. Fine-needle aspiration of the left prescapular lymph node was consistent with reactive lymphadenopathy. Complete blood count and blood gas analysis demonstrated mild leukocytosis (WBC 19.03 × 10^3^/μL; reference interval 5.05–16.76 × 10^3^/μL), hypercalcemia with an elevated ionized calcium (iCa) concentration of 1.66 mmol/L (reference interval 1.16–1.40 mmol/L) and metabolic acidosis, as evidenced by an elevated anion gap (27.3 mmol/L; reference interval 10–20 mmol/L), decreased bicarbonate concentration (HCO₃16.2 mmol/L; reference interval 21–28 mmol/L) and negative base excess (−7.0 mmol/L). Thoracic radiography revealed no evidence of pulmonary metastasis or other significant abnormalities.

The patient was fasted for a minimum of 12 h prior to imaging. Anesthesia was induced with intramuscular medetomidine (3 μg/kg; Domitor, Zoetis; Parsippany, NJ, USA) and intravenous propofol (6 mg/kg; Anepol Inj., Hana Pharm Co., Ltd.; Seoul, South Korea), followed by endotracheal intubation. Anesthesia was maintained with isoflurane (Terrell; Piramal Critical Care; Bethlehem, PA, USA) in 100% oxygen delivered via mechanical ventilation. Intravenous fluid therapy was administered at twice the maintenance rate to support blood pressure during anesthesia. Sternal recumbency was maintained throughout the imaging procedure.

Following anesthetic induction, ^18^F-FDG (0.187 mCi/kg) was administered intravenously. Whole-body computed tomography (CT) was performed using a multidetector CT scanner (100 mAs, 120 kVp, slice thickness 1.25 mm; Discovery-72 STE, General Electric Medical Systems, Waukesha, WI, USA), with pre- and post-contrast image acquisition after intravenous administration of iohexol (880 mg/kg; Omnipaque, GE Healthcare, Marlborough, MA, USA). PET was subsequently performed on the same scanner after a 60-min ^18^F-FDG uptake period, during which the patient remained under general anesthesia. The total imaging duration was approximately 2 h. Regions of interest were manually delineated on fused ^18^F-FDG PET/CT images, and the maximum standardized uptake value (SUVmax) was measured for each lesion. Image analysis was performed using RadiAnt DICOM Viewer (Medixant, Poznań, Poland).

CT identified a mass measuring 3.8 × 2.6 × 3.8 cm within the right anal sac. The internal iliac lymph node measured 3.5 × 2.2 × 1.6 cm and the lumbar aortic lymph node measured 1.3 × 1.0 × 1.3 cm (length × width × height). ^18^F-FDG PET/CT demonstrated focally increased ^18^F-FDG uptake in the right anal sac mass with an SUVmax of 7.3, the internal iliac lymph node with an SUVmax of 6.35 and the lumbar aortic lymph node with an SUVmax of 4.3 (Figure 1). No evidence of distant metastatic ^18^F-FDG uptake was identified in the lungs, liver, spleen, or other visceral organs. Based on these findings, the patient was preoperatively classified as Stage IIIa, defined as the presence of locoregional lymph node metastasis with a maximum metastatic lymph node diameter of less than 4.5 cm and no evidence of distant metastasis (11).

**Figure 1:**
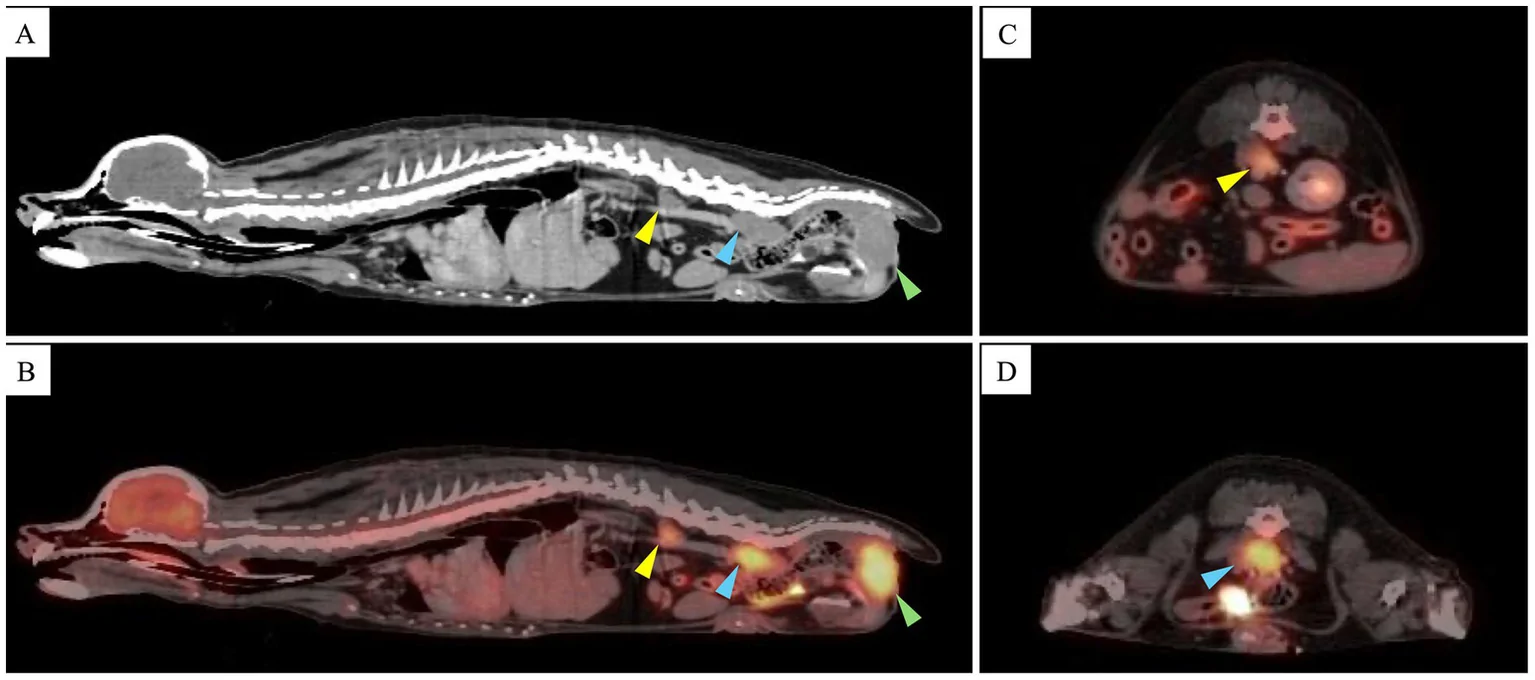
Preoperative computed tomography (CT) and ^18^F-fluorodeoxyglucose positron emission tomography/computed tomography (^18^F-FDG PET/CT) findings. Yellow arrowheads indicate the lumbar aortic lymph node, blue arrowheads indicate the internal iliac lymph node, and green arrowheads indicate the primary AGASACA mass. **(A)** Sagittal CT image showing the primary AGASACA mass and enlarged internal iliac and lumbar aortic lymph nodes. **(B)** Sagittal fused PET/CT image showing increased ^18^F-FDG uptake in the primary AGASACA mass (SUVmax 7.3), internal iliac lymph node (SUVmax 6.35), and lumbar aortic lymph node (SUVmax 4.3). **(C,D)** Transverse fused PET/CT images showing increased ^18^F-FDG uptake in the lumbar aortic lymph node and internal iliac lymph node.

The patient underwent right anal sacculectomy combined with intra-abdominal lymphadenectomy. Intra-abdominal lymphadenectomy was performed before right anal sacculectomy to minimize contamination of the abdominal surgical field. Based on the increased ^18^F-FDG uptake observed on PET/CT, the internal iliac and lumbar aortic lymph nodes were selected for surgical removal. After retraction of the abdominal viscera, the enlarged lumbar aortic lymph node was grossly observed adjacent to the aorta at the level of the caudal pole of the right kidney and was excised. The lumbar aortic lymph node was observed to consist of two distinct segments, each of which was harvested separately (part 1 and part 2). Exploration proceeded caudally, and the enlarged internal iliac lymph node was excised. During right anal sacculectomy, gross invasion of part of the rectal wall was observed. The involved portion was resected with the primary mass. All excised specimens were fixed in 10% neutral buffered formalin and submitted for histopathological evaluation (IDEXX Laboratories, Inc., USA) (Figure 2).

**Figure 2:**
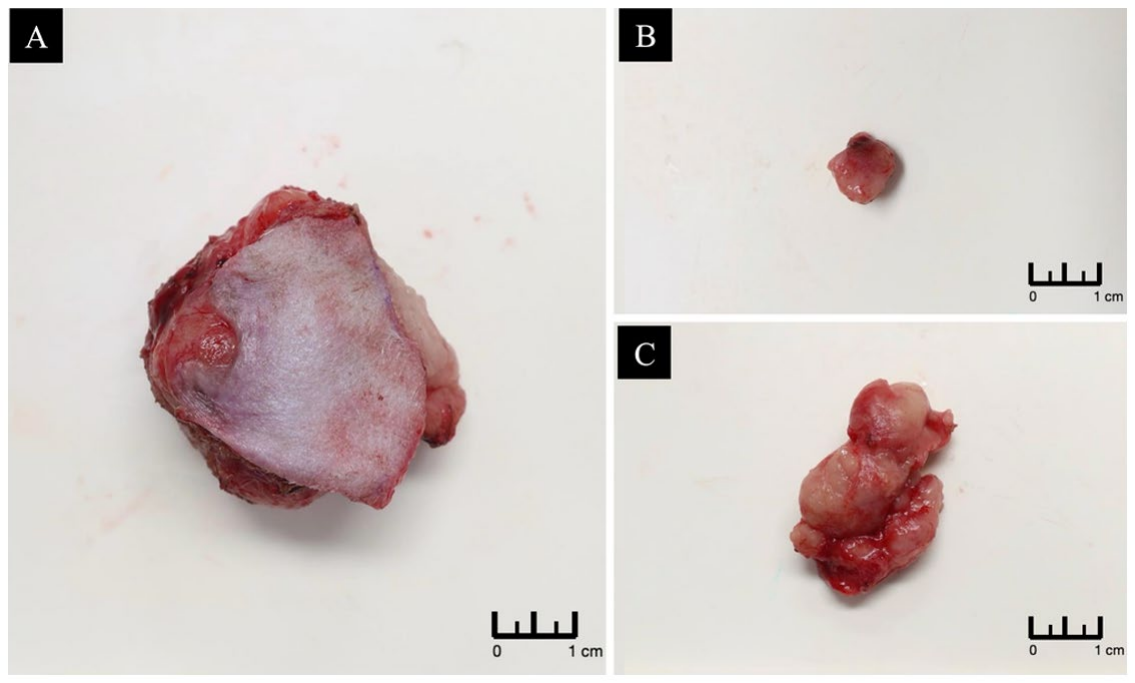
Gross appearance of the resected primary AGASACA mass and metastatic lymph nodes. **(A)** Primary AGASACA mass measuring 3.8 × 2.6 × 3.8 cm. **(B)** Resected lumbar aortic lymph node measuring 1.3 × 1.0 × 1.3 cm. **(C)** Resected internal iliac lymph node measuring 3.5 × 2.2 × 1.6 cm. (Scale bar = 1 cm).

Histopathology confirmed AGASACA, characterized by tubular to acinar differentiation, moderate anisocytosis and anisokaryosis, necrosis, and desmoplasia. The mitotic count was 30 per 2.37 mm^2^, with no vascular invasion identified in the primary mass. The lymph node lesions showed similar morphology, supporting metastasis from the primary anal sac tumor (Figure 3). Mitotic counts were 35 and 30 mitoses per 2.37 mm^2^ in parts 1 and 2 of the lumbar aortic lymph node, respectively, and 16 mitoses per 2.37 mm^2^ in the internal iliac lymph node.

**Figure 3:**
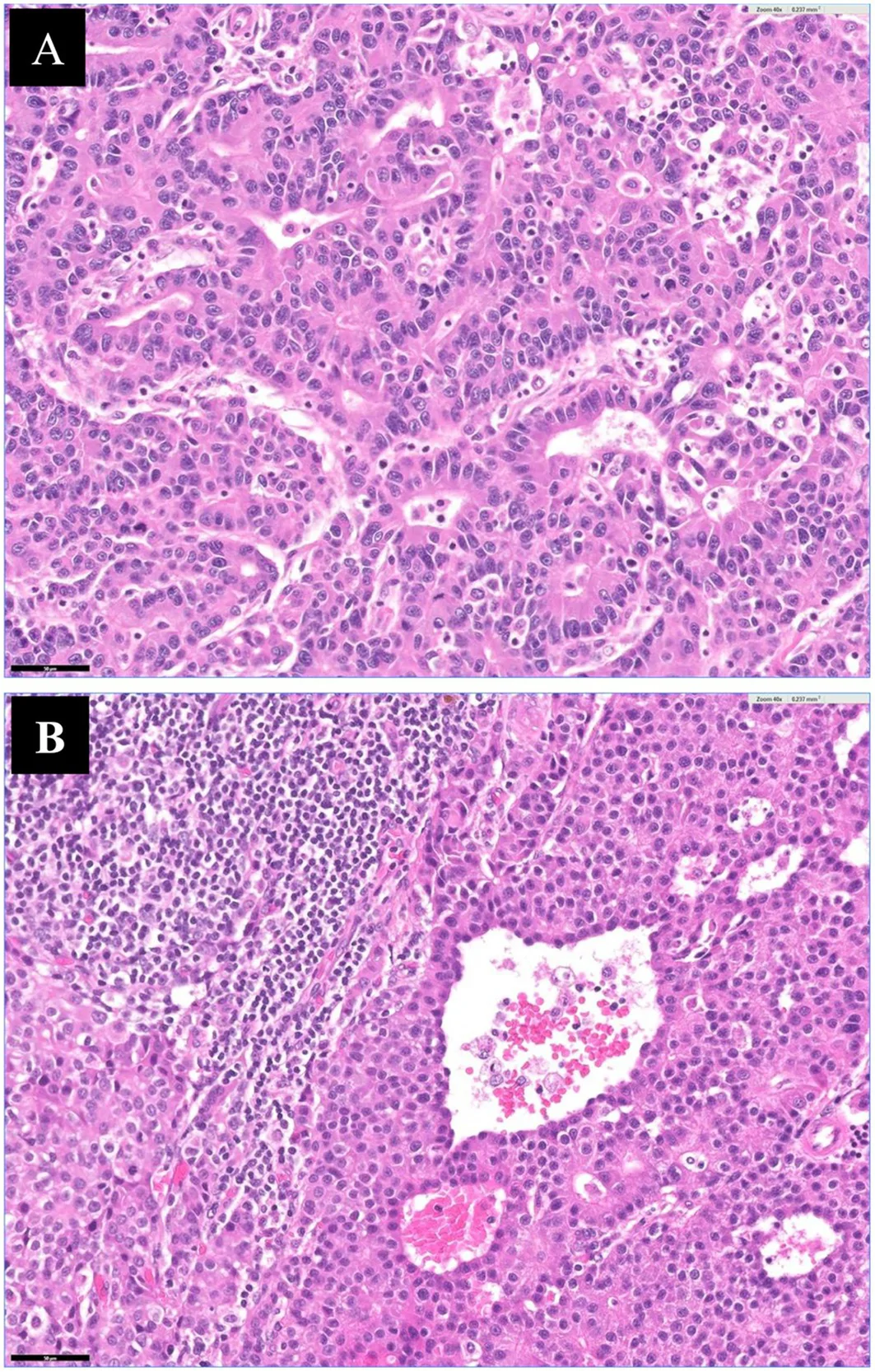
Histopathological findings of the primary AGASACA mass and metastatic lymph node. **(A)** The primary tumor comprises cuboidal to polygonal cells forming tubular and acinar structures within a desmoplastic stroma. Cells have eosinophilic cytoplasm and round to oval nuclei with prominent nucleoli and moderate anisocytosis and anisokaryosis. **(B)** In the metastatic lymph node, the normal lymphoid tissue is replaced by neoplastic cells morphologically similar to those of the primary tumor, supporting metastasis from the primary anal sac tumor. (H&E stain, original magnification×400, scale bar = 50 μm).

Postoperative treatment until discharge consisted of cefazolin (22 mg/kg IV, every 12 h; Cephazolin sodium, Chong Kun Dang Pharmaceutical Corporation., Seoul, Korea), famotidine (1 mg/kg IV, every 12 h; Gaster inj., Dong-A Pharm, Seoul, Korea). Fecal scores of 1–2 were recorded, so lactulose was administered orally at 1 mL per dog every 12 h to soften feces and reduce mechanical stress on the repair during defecation.

On the day after surgery, iCa concentration decreased to 1.35 mmol/L, falling within the reference interval, suggesting resolution of paraneoplastic hypercalcemia following resection of the primary tumor and metastatic lymph nodes. Surgical site dehiscence was noted postoperatively, and the patient underwent a secondary surgical procedure for dehiscence repair. Metronidazole (10 mg/kg IV, every 12 h; Metrynal Inj., Dai Han Pharm. Co., Ltd., Seoul, Korea) was added. The patient was subsequently discharged 12 days after the initial surgery.

Follow-up examinations were performed weekly during the first month and monthly thereafter. The owner reported that the dog had a good appetite and improvement in clinical signs after surgery. No fecal incontinence was observed by the owner. At approximately 3 months postoperatively, thoracic radiography and abdominal ultrasonography revealed enlargement of multiple intra-abdominal lymph nodes. The patient presented with signs of difficult defecation, and rectal examination identified ventral displacement of the rectum by an enlarged lymph node mass as the likely contributing factor. Hematological evaluation demonstrated recurrent hypercalcemia (2.31 mmol/L), indicative of progressive intra-abdominal lymph node metastasis and associated paraneoplastic syndrome. No evidence of distant metastasis was identified. The owner declined further tumor-directed treatment, and hospice care was provided. Thereafter, the patient experienced a decline in quality of life, including anorexia, worsening pain, and inability to ambulate, and was euthanized on postoperative day 104 (Figure 4).

**Figure 4:**
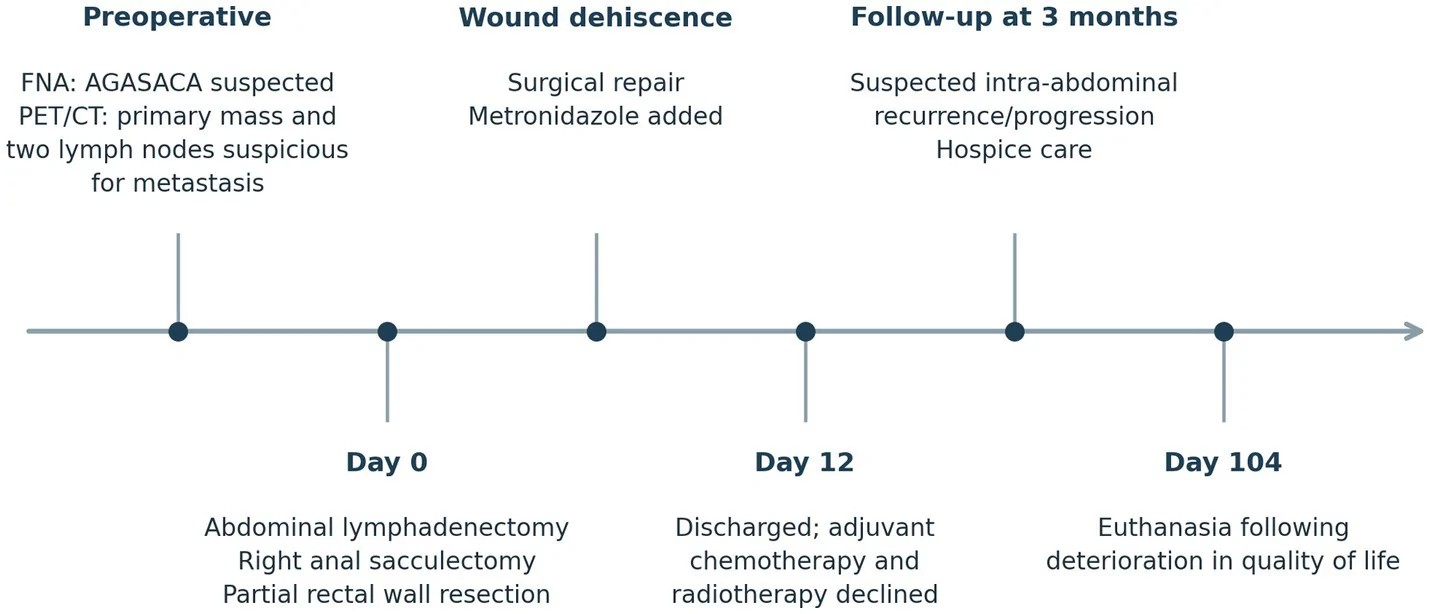
Timeline of diagnostic assessment, treatment, and follow-up. Day 0 denotes the initial surgery. Postoperative histopathology confirmed AGASACA and lymph node metastases. Recurrence and metastatic progression at approximately 3 months were suspected from the clinical and imaging findings.

## 3 Discussion

The standard staging modalities for AGASACA, abdominal ultrasonography and CT, rely on morphological and size-based criteria for lymph node evaluation and are inherently limited in their ability to provide information regarding the metabolic activity of lesions (10). FNA allows cytological confirmation but is invasive and may be technically challenging, particularly for lymph nodes situated deep within body cavities (2).

In addition, nodal staging in AGASACA may be complicated by variability in lymphatic drainage patterns, and metastatic spread may not always follow a predictable anatomical distribution. In a prospective pilot study of eight dogs with unilateral AGASACA, preoperative and intraoperative sentinel lymph node mapping findings differed in four dogs, with additional sentinel lymph nodes identified intraoperatively in several cases. Furthermore, intraoperative mapping identified sentinel lymph nodes contralateral to the primary tumor in four of eight dogs (12). Locoregional metastasis most commonly involves the iliosacral lymphocenter, whereas involvement of the lumbar aortic lymph nodes has been less commonly reported (2). In the present case, ^18^F-FDG PET/CT identified metabolically active lesions in both the internal iliac and lumbar aortic lymph nodes, and both were selected for surgical resection based on these findings. The present findings suggest that ^18^F-FDG PET/CT may complement conventional morphology-based imaging by identifying metabolically active lymph nodes at both commonly and less commonly affected nodal sites, thereby contributing to preoperative nodal staging and surgical planning in canine AGASACA.

The primary AGASACA in the present case demonstrated an SUVmax of 7.3. Because ^18^F-FDG PET/CT findings have not been systematically characterized in canine AGASACA, no disease-specific reference range or diagnostic threshold is currently available. In a study evaluating various canine malignancies, carcinomas demonstrated SUVmax values ranging from 7.6 to 27.0, including values of 12.8 and 17.1 for squamous cell carcinomas and 7.6 and 27.0 for adenocarcinomas (13). In a separate study evaluating ^18^F-FDG PET/CT in canine mammary gland tumors, malignant mammary carcinomas demonstrated SUVmax values ranging from 2.97 to 10.01, and a single case report of canine adrenocortical carcinoma reported an SUVmax of 3.24 (14, 15). Thus, although direct comparison across tumor types and studies should be interpreted cautiously, the SUVmax of 7.3 observed in the present case was comparable to values previously reported for other canine carcinomas, including adenocarcinomas.

The SUVmax values recorded for the internal iliac lymph node and lumbar aortic lymph node clearly exceeded the threshold range of 2.5–3.5 referenced as a general criterion for identifying metabolically active lymph nodes suspicious for metastasis (6). Both sites were confirmed as metastatic adenocarcinoma of AGASACA origin on postoperative histopathology, corroborating the preoperative Stage IIIa classification based on ^18^F-FDG PET/CT findings. Interestingly, the internal iliac lymph node demonstrated a higher SUVmax despite having a lower mitotic count than the lumbar aortic lymph node. This observation suggests that ^18^F-FDG uptake may not directly reflect mitotic activity alone. Although SUVmax has been positively correlated with mitotic count, FDG uptake represents a complex measure influenced by multiple biological factors, including cellular composition and glucose metabolic activity (16).

As a single case report, the ^18^F-FDG PET/CT findings described here cannot be generalized to the broader AGASACA population, and the observed SUVmax values should be interpreted with caution. Furthermore, SUVmax measurements are known to vary across institutions depending on differences in scanner hardware, image acquisition parameters and reconstruction algorithms, limiting direct comparisons between studies (13). Despite these limitations, this case describes the ^18^F-FDG PET/CT findings in a dog with AGASACA and histologically confirmed metastases to the internal iliac and lumbar aortic lymph nodes. These observations highlight the complementary metabolic information provided by PET/CT for preoperative nodal assessment in this case.

## Data Availability

The raw data supporting the conclusions of this article will be made available by the authors, without undue reservation.
